# Effects of Interferon-Alpha Treatment on the Incidence of Hyperglycemia in Chronic Hepatitis C Patients: A Systematic Review and Meta-Analysis

**DOI:** 10.1371/journal.pone.0039272

**Published:** 2012-06-29

**Authors:** Wei Zhang, Hui-Ying Rao, Bo Feng, Feng Liu, Lai Wei

**Affiliations:** Peking University People’s Hospital, Peking University Hepatology Institute, Beijing Key Laboratory of Hepatitis C and Immunotherapy for Liver Diseases, Peking University, Beijing, China; University of Modena & Reggio Emilia, Italy

## Abstract

**Background:**

There is a significant association between effects of interferon-alpha treatment and the risk of developing hyperglycemia in patients with chronic hepatitis C virus (HCV) infection. The objective of this systematic review and meta-analysis on the basis of published observational studies was to estimate risk of hyperglycemia in chronic HCV patients who had acquired sustained virological responses (SVR) compared to those without SVR.

**Methodology:**

We identified eligible studies by searching the relevant databases, including PubMed, Embase, and Google, for papers published between January 1990 and April 2011. The selection of eligible papers was carried out using a scoring system based on guidelines and inclusion criteria that were established before the articles were identified. Heterogeneity across studies was determined and the meta-analysis was performed following standard guidelines.

**Conclusions:**

Eleven eligible studies provided data of the incidence of hyperglycemia in chronic hepatitis C patients with SVR in comparison with patients without these conditions. The results demonstrated that SVR was associated with a lower risk of hyperglycemia (odds ratio = 0.497, 95% confidence interval 0.421–0.587, *p*<0.001), and there was no evidence of any substantial between-study heterogeneity (I^2^ = 24.4%, *p*>0.1). Results of meta-regression showed patients with different baseline glucose (normal vs. abnormal) and patients with co-infected HIV (presence vs. absence) as the sources of low heterogeneity (*p*<0.15).The lowest risk of hyperglycemia was described in patients with normal glucose baseline (OR = 0.402, 95%CI 0.297–0.543, *p*<0.001). This is the first systematic review and meta-analysis performed to examine the association between SVR and risk of hyperglycemia in patients with HCV infection. Our meta-analysis suggests that SVR reduce the risk of developing glucose abnormalities, especially in patients with normal glucose baseline.

## Introduction

Recent observational studies, demonstrate a significant association, but not causation, between effects of interferon-alpha treatment (e.g., sustained virological response [SVR]) and the risk of developing hyperglycemia in patients with chronic hepatitis C virus (HCV) infection. Most of the existing reports describe SVR reduce the incidence of hyperglycemia. However, others reports are on the contrary. The objective of this systematic review and meta-analysis on published observational studies was to estimate risk of hyperglycemia in chronic HCV patients who had acquired SVR compared to those without SVR (NONSVR). The targeted population of this study was defined as adults with HCV who were treated with interferon (IFN)-alpha or PEG IFN-alpha monotherapy or plus ribavirin (RBV) for 24 or 48 weeks and who were diagnosed as hyperglycemia (i.e., diabetes and/or pre-diabetes).

## Methods

### Searching of the Relevant Databases

We conducted a search of the medical literature for articles published between January 1990 and April 2011 using PubMed, Embase, and Google. The mesh-terms or key words (‘hepatitis C’ OR ‘chronic hepatitis C’ OR ‘hepatitis c virus’) AND (‘glucose’ OR ‘glucose abnormalities’ OR ‘hyperglycemia’ OR ‘prediabetes’ OR ‘diabetes mellitus’ OR ‘insulin’ OR ‘insulin resistance’ OR ‘insulin deficiency’) AND (‘interferon alpha’ OR ‘peginterferon alpha’ OR ‘interferon alfa’ OR ‘peginterferon alfa’ OR ‘IFN’) were used to obtain the search string.

### Selection

The meta-analysis was performed using summary data. No restrictions were placed on sample size, or population. When multiple reports were available for a single unique study population, we included only the most recent or largest report.

Because bias in observational studies is a problem, we perform the analysis (data permitting) to verify suspected sources of bias and variability in the study findings [1], [2].

Eligible studies met the following criteria. 1) They were designed as cohort studies. 2) They involved chronic HCV patients who were treated with IFN- alpha or PEG IFN-alpha monotherapy or plus ribavirin for 24 or 48 weeks. 3) They included expose (SVR) or unexposed (NONSVR) groups. 4) They included data on the incidence rate of hyperglycemia.

Studies were excluded if they met any of following criteria: 1) were not designed to discuss the key question; 2) were not published as original articles (including letters, abstracts, reviews and editorials); 3) were not published in English; 4) included children, dialysis patients, pregnant women, or patients who had undergone transplantation or been diagnosed with ketoacidosis, diseases of the exocrine pancreas, any other endocrinopathies, or cancer.

### Validity Assessment

To determine the quality of each study, we created a scoring system based on the guidelines developed by Moose [1], Quatso [3], and Strobe [4]. We assessed five characteristics: 1) targeted population as adults with chronic HCV (1 point if the diagnosis was made by detecting either anti-HCV or HCV RNA [5]); 2) clear definition of exposures (SVR) (1 point if SVR was defined as the number of patients with detectable HCV RNA in serum by sensitive testing within 24 weeks after the end of IFN-alpha treatment); 3) clear definition of outcomes (incidence of hyperglycemia including diabetes and/or pre-diabetes) (1 point if diagnosis of the normal glucose group, diabetes mellitus and pre-diabetes was in accord with American Diabetes Association [ADA] guidelines [6]); 4) study design (1 point if any justification was given for the cohort and 1 point for appropriate inclusion and exclusion criteria); and 5) statistical analysis (1 point was given if adjustments were made for age, body mass index (BMI),family history of type 2 diabetes, etc. as proven risk factors for the development diabetes and pre-diabetes, and 1 point was given if adjustments were made for SVR risk factors). Studies were graded as “good quality” if they met at least six of seven points, and as “poor quality” if they met fewer than four points.

### Data Abstraction

All of the eligible articles were reviewed by pairs of researchers; each pair included at least one reviewer with clinical training and one with training in epidemiology and research methods. One reviewer completed the quality assessment and data-extraction forms. Disagreements about eligibility were resolved by consensus. The following information was extracted from each study included in the current analysis: the number of patients with hyperglycemia (diabetes and/or pre-diabetes), hazard ratio (HR) or odds ratio (OR) estimating the association, study design, sample size, and participant characteristics. Characteristics of patients, including age, sex, cirrhosis, glucose baseline status, co-infected with human immunodeficiency virus (HIV), ethnics, high BMI, family history of diabetes, steatosis, homeostasis model assessment for insulin resistance (HOMA-IR) values, ALT levels, HCV-RNA, HCV genotype, liver fibrosis, naïve and experienced patients, types of DM, alcohol consumption, and treatment schedules, were recognized as confounding factors.

### Quantitative Data Synthesis

Our primary goal was to assess the risk of hyperglycemia in chronic HCV patients who had acquired SVR compared to those without SVR. The relative risk was the ratio of the rate of the index subjects to that of the control subjects. The OR was generally a good estimate of the relative risk.

We used two models of meta-analysis (the random-effects model and the fixed-effects model) to evaluate a summary estimate of the overall association between SVR and hyperglycemia in adults with chronic HCV. Heterogeneity was considered significant for *p* value of Cochran’s Q statistic <0.10 and I^2^>50% [7], [8]. I^2^ was the percentage of variation attributed to heterogeneity and was easily interpreted. Higgins et al. tentatively suggested I^2^ values between 25–50% be considered low, 50–75% be considered moderate, and ≥75% be considered high. Our decision to perform fixed-effects analysis or random-effects analysis was based on the results of the heterogeneity assessments. As all tests showed great heterogeneity, random effects models were preferred to pool the results. On the contrary, fixed effects models were chosen.

Exploring the possible sources of heterogeneity between studies was an important aspect of conducting a meta-analysis. Meta-regression was applied to investigate the heterogeneity of the studies [9]. According to the widely accepted minimum sample size for regression analysis [10], we performed meta-regression only when there were ≥10 comparable studies. Variables significant at *p*<0.15 were considered potentially important sources of between-study heterogeneity. A meta-regression was performed involving confounding factors (i.e., baseline glucose, co-infected HIV, cirrhosis, and treatment schedules), and study characteristics (i.e., publication year, publication area, study design, sample size, and quality score). We performed stratification analyses according to baseline glucose (normal vs. abnormal), presence vs. absence of cirrhosis, treatment schedules (PEG IFN-alpha-2b/2a vs. IFN-alpha-2b/2a), publication area, and study design.

A sensitivity analysis was performed by the sequential omission of individual studies as a possible major source of heterogeneity.

Publication bias was investigated by Begg’s funnel plots and Egger’s regression asymmetry test [11], [12]. Funnel plot shapes did not reveal obvious evidence of asymmetry, and all the *p* values of Egger’s tests were more than 0.05, providing statistical evidence of the funnel plots symmetry.

In this meta-analysis, if there was no event in one group, 0.5 was added to each cell for these calculations [13].

Statistical analyses were conducted using STATA 12.0.

## Results

### Flow of Included Studies

**Figure 1:**
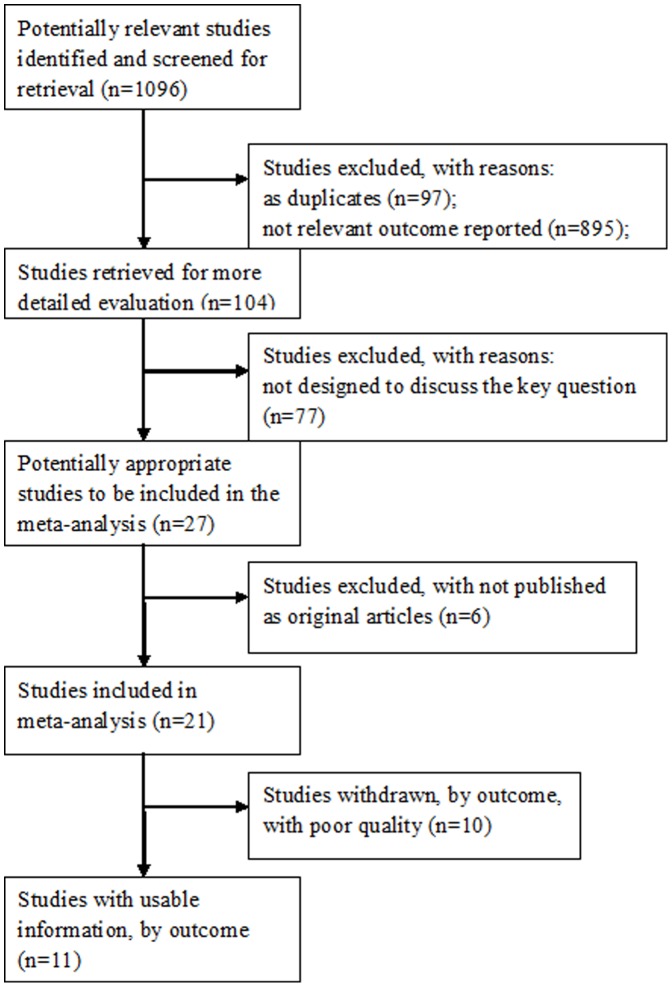
Flow of included studies. The reasons for exclusion included: no relevant outcome reported (n = 895); duplicates (n = 97); not designed to discuss the key question (n = 77); not considered an original publication or research (i.e., letters, abstracts, reviews, editorials, etc.) (n = 6); poor quality (n = 10, including studies containing data on patients that were not in accordance with our diagnostic criteria (n = 9); or the study was not designed as a cohort study (n = 1)). Therefore, two prospective cohort studies and nine retrospective cohort studies were included in our meta-analysis.

The literature search identified 1096 English abstracts. After reviewing the abstracts, 992 reports were excluded. Relevant full-text publications (n = 104) were identified for further detailed evaluation, and 11 studies were identified as providing useful information (1%). The most frequent reasons for exclusion included: no relevant outcome reported (n = 895); duplicates (n = 97); not designed to discuss the key question (n = 77); not considered an original publication or research (i.e., letters, abstracts, reviews, editorials, etc.) (n = 6); poor quality (n = 10, including studies containing data on patients that were not in accordance with our diagnostic criteria (n = 9); or the study was not designed as a cohort study (n = 1)). Therefore, two prospective cohort studies and nine retrospective cohort studies were included in our meta-analysis. We identified available data from two unique studies in two publications [14], [15] (Figure 1).

### Study Characteristics

The main characteristics of these studies are described in Table 1. They were all published between 2006–2010. The sample size varied between 51–1059 patients. More than half of these studies were performed in Europe (n = 6) and the others were performed in Asia (n = 5). Among these, nine studies were performed in one hospital, and two studies were multicenter trials (11 hospitals). Eight studies provided respective data on diabetes mellitus (DM) or pre-diabetes [14]–[19] and other three studies reported total data on DM and pre-diabetes [20]–[22].Only one study [20] reported data in patients co-infected with HIV.

**Table 1:** Characteristics of meta-analysis eligible studies examining the association between SVR and Hyperglycemia.

Study	Year	Country	Design	Hyper/SVR	NOR/SVR	Hyper/NONSVR	NOR/NONSVR	Sample	OR/HR
**Simo, R. ** [**16**]	2006	Spain	retrospective	14	82	47	91	234	HR0.489(0.278–0.890), *p* = 0.018;
**Lecube, A. ** [**17**]	2007	Spain	retrospective	16	51	49	62	178	OR 2.72(1.12–6.59), *p* = 0.026;
**Giordanino, C. ** [**18**]	2008	Italy	prospective	12	81	21	88	202	HR0.88(0.38–2.02), *p* = 0.758;
**Romero-Gomez,M.-1 ** [**14**]	2008	Spain	retrospective	143	432	182	302	1059	OR 0.44(0.20–0.97), *p* = 0.04;
**Romero-Gomez,M.-2 ** [**14**]	2008	Spain	prospective	50	382	74	228	734	NR
**Cesari, M. ** [**20**]	2009	Italy	retrospective	7	44	21	24	96	OR0.133(0.034–0.512), *p* = 0.003;
**Chehadeh, W. ** [**21**]	2009	Kuwait	retrospective	34	48	36	41	159	OR 8.50(7.1–14.6), *p*<0.001
**Kawaguchi, Y.-1 ** [**15**]	2009	Japan	retrospective	14	34	10	14	72	*p* = 0.335;
**Kawaguchi, Y.-2 ** [**15**]	2009	Japan	retrospective	5	40	7	13	55	NR
**Konishi, I. ** [**19**]	2009	Japan	retrospective	30	62	42	64	197	NR
**Mizuta, T. ** [**22**]	2010	Japan	retrospective	6	17	11	17	51	*p* = 0.38;

Hyper/SVR: the risk of hyperglycemia in CHC patients who acquired sustained virological responses; NOR/SVR: data of normal glucose in CHC patients who acquired sustained virological responses; Hyper/NONSVR: the risk of hyperglycemia in CHC patients who did not acquire sustained virological responses; NOR/NONSVR: data of normal glucose in CHC patients who did not acquire sustained virological responses; OR: odds ratio; HR: hazard ratio; NR: not reported.

Table 2 shows a brief description of the patients included in the meta-analysis. The mean patient age was 47.6 years (n = 9), ranging between 42.8–54.7years. The average BMI of these studies was 24.2 kg/m^2^ (n = 9). The percentage of females was 37.22%, ranging between 15.6–38.7%. The proportions of patients with normal glucose baseline were 40.34%. Of these studies, two did not report the proportion of HCV genotype1 [15], [21]. The prevalence rate of infection with genotype 1 was 72.55%, ranging between 37.5–100%. Of these studies, 10 reported data on the proportion of patients with cirrhosis (17.14%). The mean alanine aminotransferase (ALT) of all patients was 97.3 U/L(n = 9), ranging between 69.73–114.36 U/L. Only Asians (12.34%) were reported in these studies (n = 4). The proportions of patients administered IFN treatment was as follows: 25.55% were on IFN-α-2b/2a+RBV, 9.14% were on IFN-α-2b/2a alone, and 60.96% were on Peg IFN-α-2a/2b.

**Table 2:** Characteristics of patients in the meta-analysis.

Characteristics	Number of patients	Percent of patients
**Age (means 47.6 years)**	2823	92.95%(2823/3037)
**BMI (means 24.2 kg/m^2^)**	2823	92.95%(2823/3037)
**Female**	1110	37.22%(1110/2982)
**Genotype1**	2048	72.55%(2048/2823)
**With normal glucose baseline**	1225	40.34%(1225/3037)
**With HIV**	96	3.16%(96/3037)
**With cirrhosis**	490	17.14%(490/2859)
**With retreatment**	105	23.23%(105/452)
**With DM treatment**	12	0.47%(12/2542)
**ALT (means 97.3U/L)**	2780	91.54%(2780/3037)
**IFN-alpha −2b/2a+RBV treatment**	604	25.55%(604/2364)
**IFN-alpha −2b/2a alone**	216	9.14%(216/2364)
**PEG IFN-alpha −2b/2a+RBV**	1441	60.96%(1441/2364)

BMI: body mass index; HIV: human immunodeficiency virus; DM: diabetes mellitus; ALT: alanine aminotransferase; IFN: interferon; RBV: ribavirin.

### Quantitative Data Synthesis

**Figure 2:**
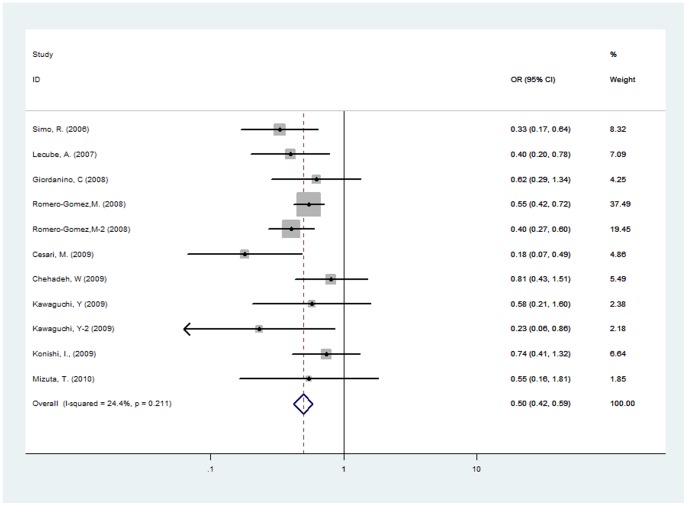
ORs and 95% CI of the association between SVR and hyperglycemia. SVR was associated with a lower risk of hyperglycemia in chronic HCV patients (OR = 0.497; 95% CI 0.421–0.587). There was no evidence of substantial between-study heterogeneity.

Eleven studies involving 3037 patients were included in the meta-analysis. There was no evidence of substantial between-study heterogeneity (I^2^ = 24.4%, *p*>0.1); thus, the fixed-effects model was used to pool the results. The results demonstrate that SVR is associated with a lower risk of hyperglycemia in chronic HCV patients (OR = 0.497, 95% CI 0.421–0.587, *p*<0.001) (Figure 2).

It is well known that performing meta-regression in order to explore sources of heterogeneity is appropriate, even if an initial overall test for heterogeneity in insignificant [9], [23]. Meta-regression was applied to determine the heterogeneity of this meta-analysis. Study characteristics and confounding factors were investigated by univariate meta-regression (n≥10). The study including co-infected HIV patients [20] was significantly identified as sources of heterogeneity (*p*<0.15). After excluding this study, the incidence of hyperglycemia was 51.3% (OR = 0.513, 95%CI 0.434–0.608, *p*<0.001) (I^2^ = 1.1%, *p*>0.1). Among confounding factors, baseline glucose was showed as the source of heterogeneity (*p*<0.15).

Results in stratification analyses were as follows. The lowest risk of hyperglycemia was described in patients with normal glucose baseline (OR = 0.402, 95%CI 0.297–0.543, *p*<0.001). And the risk in patients with abnormal glucose before IFN-alpha treatment was higher compared to those with normal glucose baseline (OR = 0.547, 95%CI 0.448–0.668, *p*<0.001). The highest risk was reported in Asian (OR = 0.597, 95%CI 0.385–0.925, *p* = 0.02). However, there was no significant difference between SVR and NONSVR groups in Asian. In comparison with patients without cirrhosis, cirrhosis patients had higher OR (0.528, 95%CI 0.440–0.634, *p*<0.001). In retrospective studies, the incidence of hyperglycemia was 51.4% (95%CI 0.426–0.621, *p*<0.001) (I^2^ = 31.3%, *p*>0.1).

### Sensitivity and Publication Bias

Sensitivity analysis was performed by sequential omission of individual studies. For analyses that included more than three pooled individual studies, the significance of the OR was not significantly altered by omitting any single study (data not shown).

**Figure 3:**
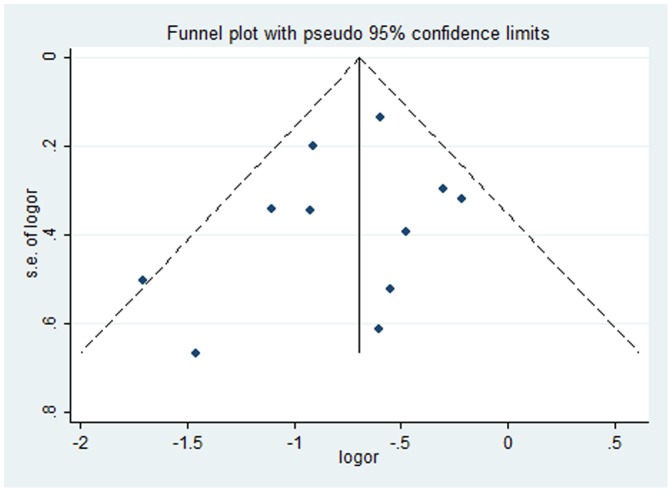
Funnel plot. The funnel plot’s shape is in asymmetrical. There was no significant publication bias indicated in the main analysis.

Among 11 studies that estimated the association between SVR and the incidence of hyperglycemia, no evidence of publication bias was found in the main analysis *(p*>0.05) (Figure 3).

### Study Quality

Ten studies (90.9%) were identified as good quality (i.e. ≥6 points). Six were missing a point for statistical analysis (6 points). Four studies met all of the criteria of the quality- assessment tool, and some adjustments were made for potential confounding factors, such as age, BMI, family history of diabetes, HOMA-IR, HCV genotype, HCVRNA levels, AST, ALT, and cirrhosis.

## Discussion

To the best of our knowledge, this is the first systematic review and meta-analysis performed to examine the association between SVR and risk of hyperglycemia (DM and/or pre-diabetes) in patients with HCV infection on the basis of published observational studies. Our meta-analysis, which was carried out on eligible studies, suggests that SVR reduce the risk of developing glucose abnormalities. Accordingly, NONSVR patients are at an almost two-fold greater risk of developing glucose abnormalities compared with SVR patients.

We found no significant evidence of statistical heterogeneity in this meta-analysis. However, non-significant statistical heterogeneity could not be interpreted as evidence of homogeneity of results of all included studies [24]. It is because tests of heterogeneity have low power and might fail to detect as statistically significant even a moderate degree of genuine heterogeneity [25]. There was the low degree of statistical heterogeneity in our meta-analysis (I^2^<50%). The existence of clinical or methodological heterogeneity would be expected to lead to at least some degree of statistical heterogeneity in the results. However, it must be ascertained that there are not substantial clinical heterogeneity of all included studies and it is appropriate to pool them prior to analysis. When combining observational studies, some heterogeneity of design, populations, and outcome is not avoided [1]. In this review, the participants were defined as adults with HCV who were treated with IFN-alpha or PEG IFN-alpha treatment and who were diagnosed as hyperglycemia. Other characteristics of patients (i.e., age, sex, cirrhosis, glucose baseline status, co-infected HIV, ethnics, high BMI, family history of diabetes, steatosis, HOMA-IR values, ALT levels, HCV-RNA, HCV genotype, liver fibrosis, naïve and experienced patients, types of DM, alcohol consumption, and treatment schedules) were recognized as confounding factors caused by the selection bias. However, the original studies do not provide enough data related to these factors. Our proper attention was given to factors, such as baseline glucose, co-infected with HIV, cirrhosis, and treatment schedules.

Meta-analyses of observational studies, these are recognized as non-randomized studies [2], present particular challenges because of inherent biases. Ozminkowski RJ, et al. addressed selection bias by aggregating the results of eligible studies according to birth weight in the meta-analysis of 19 non-randomized studies and found selection bias (infants at these birth weights) was a major factor in the explanation of the significant heterogeneity [26]. Similarly, we collected and analyzed data of outcomes of included studies according to baseline glucose, cirrhosis, co-infected HIV, and treatment schedules. Results of meta-regression showed patients with different baseline glucose (normal vs. abnormal) and patients with co-infected HIV (presence vs. absence) as the sources of heterogeneity (*p*<0.15). There was selection bias in this meta-analysis. ‘Adjusted’ estimates were accomplished through the use of stratification analysis. SVR was associated with a lower risk of hyperglycemia in chronic HCV patients, especially in patients with normal glucose baseline. Results in patients without infected HIV showed that the OR was not significantly altered.

Other confounding factors might be potential sources of heterogeneity. Unfortunately, we could not get enough data to analysis and provided evidences for them. Simo, et al. [16] demonstrated that after adjusting for the recognized predictors of both type 2 diabetes and SVR (e.g., age, BMI, AST, ALT, fibrosis, genotype, and duration of treatment), the OR for hyperglycemia in patients with SVR is 0.48(95% CI 0.24–0.98, *p* = 0.04) compared with NONSVR patients. The results of our meta-analysis do not contradict these findings. However, Giordanino, et al. [18] showed the incidence of DM and pre-diabetes was not significantly different between SVR and NONSVR patients after adjusting for baseline risk factors of DM and the predictors of a poor response. In such situations, these risk factors can be considered only as hypotheses for evaluation in future studies.

We found no evidence of publication bias, as the result in statistical analyses. There were no restrictions placed on sample size, and population in our meta-analysis. Considering selected published studies based on original articles and language restrictions, we could not ignore publication bias.

Publication bias clearly is a major threat to the validity of any type of review. Obviously, including data from unpublished studies appears to be one way of avoiding this problem. However, the inclusion of data from unpublished studies can itself introduce bias. Unpublished studies may be of lower methodological quality than published studies [27], [28]. We also exclude studies published in abstract form as following reasons. First, only about half are published as a full manuscript [29], [30]. Many abstracts are recognizes as grey literatures and might be of lower quality [31]. Their inclusion will compromise the validity of a meta-analysis. Second, abstracts could not provide enough information. This could again bias the findings. If enough information of patients in abstracts cannot be obtained, the search may be futile. Third, abstracts do not escape publication bias [31]. Abstracts with positive results tend to be accepted more frequently than those with negative findings at conferences.

Our meta-analysis was based on studies published in English. Language bias could be introduced. The effects of language bias might be diminished recently because of more and more studies published in international English-language journals [32].

There are some possible limitations to our meta-analyses. First, the number of eligible studies is small. It leads to a lack of data on some confounding factors that may influence the accuracy of the results. Second, there is potential bias in the meta-analysis of observational studies. Despite these limitations, our meta-analysis suggests that SVR be associated with a low incidence of hyperglycemia, especially in patients with normal glucose baseline. More large-scale cohort investigations that include data on the risk factors of hyperglycemia and SVR are needed to provide the evidence of a possible association.

In conclusion, SVR reduce the risk of developing glucose abnormalities, especially in patients with normal glucose baseline. The results suggest that abnormal baseline glucose as the significant risk factor and it is important to screen glucose levels in patients before IFN-alpha treatment.
